# Effects of L-Cysteine and γ-Aminobutyric Acid Treatment on Postharvest Quality and Antioxidant Activity of Loquat Fruit during Storage

**DOI:** 10.3390/ijms241310541

**Published:** 2023-06-23

**Authors:** Huifen Zhang, Jing Pu, Han Liu, Miao Wang, Ying Du, Xiaofu Tang, Xian Luo, Yongqing Wang, Qunxian Deng

**Affiliations:** College of Horticulture, Sichuan Agricultural University, Chengdu 611130, China; hfcheung@sicau.edu.cn (H.Z.); pg7191127@163.com (J.P.); 13088016648@163.com (H.L.); wangmiao202102@163.com (M.W.); yj18987246849@163.com (Y.D.); tangxf152@163.com (X.T.); lawxian@aliyun.com (X.L.); yqw14@sicau.edu.cn (Y.W.)

**Keywords:** loquat, L-Cysteine, γ-aminobutyric acid, fruit quality, antioxidant system, cold storage, shelf life

## Abstract

Sichuan is the China’s leading producer of loquat, with the largest cultivation area and yield ranked first in China. Loquat is a seasonal fruit highly appreciated by consumers; however, the fruit is prone to browning and lignification after harvest, affecting its storage quality. The effects of L-Cysteine (L-Cys, 0.01, 0.05, 0.1, 0.2%) and γ-aminobutyric acid (GABA, 0.025, 0.05, 0.075, 0.1%) on the sensory quality and antioxidant activity of loquat fruit during cold storage at 4 °C for 35 days and simulated shelf life for 5 days were investigated. The results showed that after 40 days of storage, compared with the control, 0.05% L-Cys and 0.05% GABA treatment of ‘Zaozhong No. 6’ loquat fruit effectively reduced the weight loss rate, browning index, decay index, respiratory rate, firmness, and lignin content and slowed the decreases in total soluble solids, soluble sugar, titratable acidityand vitamin C contents. The application of 0.05% L-Cys and 0.05% GABA significantly increased the contents of total phenols, total flavonoids, flavanols, and carotenoids; delayed the increase of relative electric conductivity, MDA, POD, and PPO activities; and significantly enhanced the activities of SOD and CAT, DPPH free radical scavenging ability, and FRAP, thereby improving antioxidant capacity. In summary, 0.05% L-Cys and 0.05% GABA treatment promotes the quality of loquat fruit after 40 days of storage, and significantly enhances antioxidant capacity, thus delaying senescence after harvest.

## 1. Introduction

Loquat (*Eriobotrya japonica* Lindl.) is a subtropical tree native to China. The non-climacteric fruit is soft and juicy, with a unique flavor and rich in a variety of nutrients and functional components, such as carotenoids, phenols, and other bioactive components. According to traditional Chinese medicine, the fruit has the effects of moistening the lung, relieving thirst, and clearing heat [1], and it is becoming increasingly popular among consumers; however, it is difficult to transport the fruit to market. The fruit is harvested at a time of year when the temperature is high and the weather is often rainy. With thin fruit skin, mechanical damage to loquats can result in rot and deterioration [2]. Previous studies tested treatments with chitosan coating [3], different biomaterials [4], heat treatment [5], and 1-MCP [6] to promote the synthesis of stress-resistant substances by inducing changes in the secondary metabolic pathways of the fruit, thereby delaying fruit senescence and maintaining fruit quality. Recently, natural amino acids have been widely applied in agricultural production as safe exogenous treatments. Exogenous amino acid treatment can regulate growth and development and can improve the quality of fruits and vegetables [7] and enhance the stress resistance of fruits [8].

Treatment with L-Cysteine (L-Cys), a thiol compound, can have anti-browning and anti-oxidation effects [9], which can alter plant growth and development [10]. L-Cys can react with quinones produced by enzymatic reactions to inhibit the formation of browning [11]. L-Cys can directly act on polyphenol oxidase (PPO) to inhibit enzymatic browning by reducing its activity [12]. Zhou et al. [13] found that 0.8 mg·mL^−1^ L-Cys could prevent enzymatic browning during pear-juice processing, and Wu et al. [14] found that L-Cys can reduce the browning of fresh wet noodles by reducing pH, inhibiting PPO activity and polyphenol oxidation. L-Cys can significantly slow the browning of fresh-cut lettuce and greatly improve antioxidant capacity [15]. Numerous studies have shown that exogenous L-Cys can effectively regulate the enzymatic browning of fresh-cut fruits and vegetables. Nevertheless, little is known about the role of L-Cys on loquat quality during storage.

γ-aminobutyric acid (GABA) is a four-carbon, non-protein amino acid that is widely found in animals and plants, and has important effects on plant growth and resistance [16]. GABA is a natural compound that is considered safe and may be beneficial to human health [17]. Studies have shown that GABA treatment increased the content of proline and endogenous GABA and enhanced the activity of Δ^1^-pyrroline-5-carboxylate synthetase (P5CS), ornithine δ-aminotransferase (OAT) and glutamate decarboxylase (GAD) ) to effectively limit chilling injury of peach fruit [18]. Wang et al. [19] found that 20 mM GABA treatment effectively delayed the increase of CI index and MDA content in banana peel, promoted the accumulation of total phenols and proline, and enhanced the antioxidant capacity of banana fruit. Another study on kiwifruit found that GABA-treated kiwifruit fruit had higher levels of AsA, which effectively protected against cell membrane damage [20].

Loquat cultivation has great commercial and economic value. However, the delicate fruit is at risk for quality deterioration due to its susceptibility to mechanical damage, decay, and lignification. Based on previous reports, exogenous L-Cys and GABA treatments can reduce CI, retard browning, and enhance antioxidant capacity of fruits and vegetables, thus maintaining fruit quality traits during storage, but the effects of exogenous L-Cys and GABA treatments on loquat storage quality have not been reported. Therefore, this study aimed to evaluate the effects of these treatments on postharvest quality and antioxidant capacity of loquat fruits during cold storage, to assess its application to loquat storage to improve post-harvest quality, and to provide theoretical support for postharvest preservation of loquat.

## 2. Results

### 2.1. Effects of L-Cys and GABA Treatments on Weight Loss Rate, Browning Index, Decay Index, and Respiration Intensity

Fruit weight loss rate is an important index to evaluate fruit freshness. As shown in Table 1, the weight loss rate of loquat fruit increased during storage. After 21 days of storage, the weight loss rates of L-Cys and GABA groups were significantly lower than that of the control group, with the most significant results for the 0.05% L-Cys and 0.05% GABA treatment. At the end of storage, the weight loss rates of loquat fruit treated with 0.05% L-Cys and 0.05% GABA were significantly lower, by 17.5% and 13.5%, respectively, than that of the water control (*p* < 0.05). This showed that these two exogenous treatments could inhibit the increase in weight loss rate of loquat fruit.

Throughout storage, the browning index of loquat fruit gradually increased (Table 1). After 7 days of storage, the browning index of the control group was significantly higher than those of the L-Cys and GABA treatments (*p* < 0.05), and the browning index was significantly lower for the 0.05% L-Cys and 0.05% GABA treatments (*p* < 0.05). After 40 days of storage, the browning index of the control group was 71%, while those for the 0.05% L-Cys and 0.05% GABA treatments were 49.83% and 48.17%, respectively. Thus, these two treatments acted to inhibit the browning of loquat fruit.

Decay index is an important index of the effect of fruit storage and preservation. Loquat fruit can easily rot during storage, reducing its quality. With the extension of storage time, the decay index of loquat fruit increased (Table 1). After 40 days of storage, the decay index of loquat fruit in the control group was 49.36%, while the decay index values of loquat fruit in the 0.05% L-Cys and 0.05% GABA treatment groups were 34.17% and 34.44%, respectively. These decay index values were significantly lower than that of the control group (*p* < 0.05), indicating that these two treatments effectively inhibited the decay of loquat fruit during storage.

The respiration rate of loquat fruit decreased during storage, as shown in Table 1. After 7 days of storage, the respiration rate of the control group was significantly higher than those of the L-Cys and GABA treatments (*p* < 0.05). Both treatments inhibited the respiration of the fruit, thereby delaying fruit senescence, with 0.05% L-Cys and 0.05% GABA treatments exhibiting the most significant inhibitory effects on respiration rate. Again, these results show that L-Cys and GABA treatments can effectively inhibit fruit respiration and delay the decomposition and consumption of organic matter.

### 2.2. Effects of L-Cys and GABA Treatments on TSS, SS, TA, and Vitamin C Content

**Figure 1:**
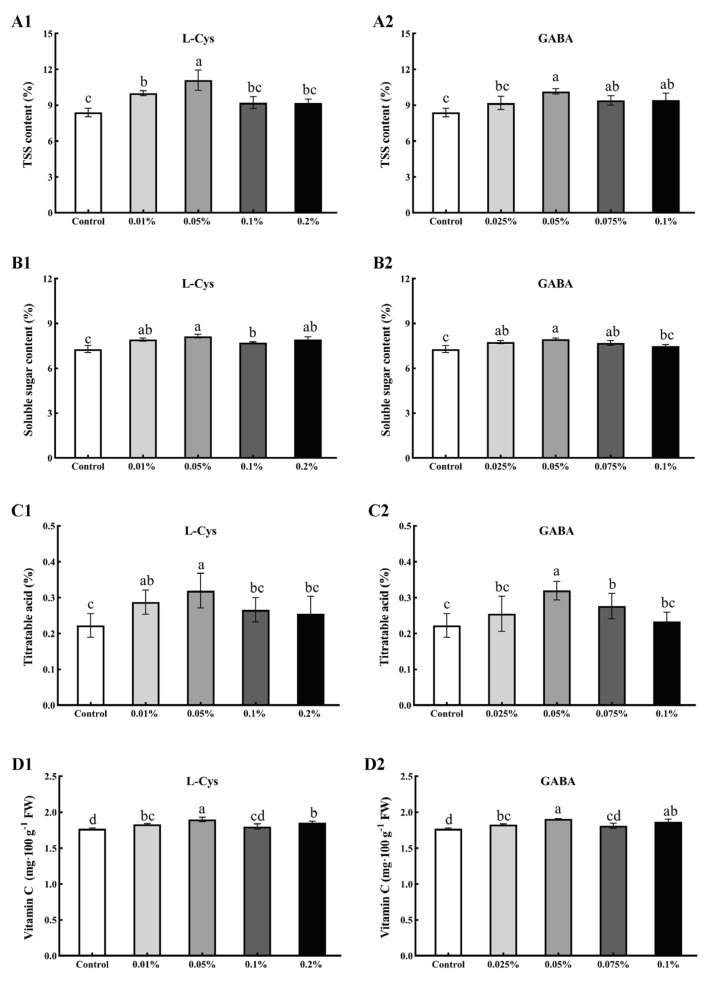
Effects of different concentrations of L-Cys and GABA treatments on TSS (**A**), SS (**B**), TA (**C**), and vitamin C content (**D**) of loquat fruit after 40 days of storage. A1, B1, C1, D1 and A2, B2, C2, D2 indicate the effect of L-Cys and GABA, respectively. Each column represents mean ± standard deviation (n = 3), and a *t* test was performed at the 0.05 significance level (*p* < 0.05). Different letters (a, b, c, d) indicate significant differences, and the same letter indicates no significant difference.

As shown in Figure 1, loquat fruits treated with 0.05% L-Cys and 0.05% GABA had significantly higher TSS, SS, TA, and vitamin C levels compared to the water control group (*p* < 0.05). Specifically, the TSS levels (Figure 1A) were 32.02% and 20.95% higher for the 0.05% L-Cys and 0.05% GABA groups, respectively, while soluble sugar levels (Figure 1B) were 11.80% and 9.05% higher, and the titratable acid content (Figure 1C) was 43.49% and 43.56% higher, respectively. The vitamin C content (Figure 1D) was 7.34% and 7.91% higher, respectively, while the other L-Cys and GABA treatment groups did not show significant differences but were significantly higher than the control group. Additionally, after 40 days of storage, the application of L-Cys and GABA significantly inhibited the decrease in TSS, SS, TA, and vitamin C content.

### 2.3. Effects of L-Cys and GABA Treatments on Firmness and Lignin Content of Loquat Fruit after Harvest

**Figure 2:**
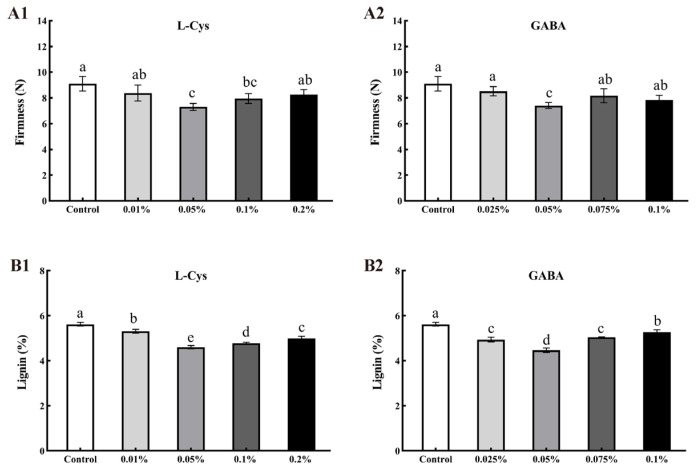
Effects of different concentrations of L-Cys and GABA treatments on fruit firmness (**A**) and lignin (**B**) of loquat fruit after 40 days of storage. A1, B1 and A2, B2 indicate the effect of L-Cys and GABA, respectively. Each column represents mean ± standard deviation (n = 3), and a *t* test was performed at the 0.05 significance level (*p* < 0.05). Different letters (a, b, c, d) indicate significant differences, and the same letter indicates no significant difference.

The trends of firmness and lignin content were consistent during loquat storage, and L-Cys and GABA treatments delayed the increase of firmness and lignin in postharvest loquat fruits. The firmness (Figure 2A) and lignin (Figure 2B) content of loquat fruits treated with 0.05% L-Cys and 0.05% GABA were the lowest, and significantly lower than those of the control (*p* < 0.05). Firmness decreased by 24.49% and 22.80%, and lignin content decreased by 22.10% and 25.54% for the 0.05% L-Cys and 0.05% GABA treatments, respectively, indicating that L-Cys and GABA treatments effectively slowed the lignification and senescence of loquat fruits during storage.

### 2.4. Effects of L-Cys and GABA on Relative Electric Conductivity and MDA Content of Loquat Fruit after Harvest

**Figure 3:**
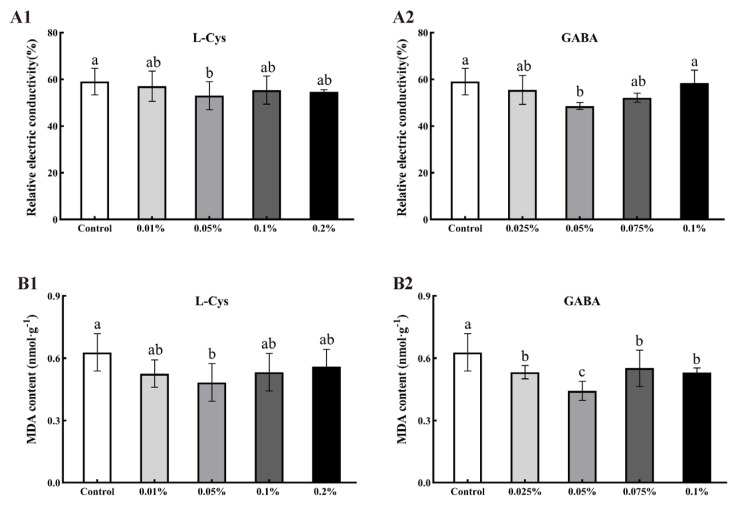
Effects of different concentrations of L-Cys and GABA treatments on relative electric conductivity (**A**) and MDA content (**B**) of loquat fruit after 40 days of storage. A1, B1 and A2, B2 indicate the effect of L-Cys and GABA, respectively. Each column represents mean ± standard deviation (n = 3), and a *t* test was performed at the 0.05 significance level (*p* < 0.05). Different letters (a, b, c) indicate significant differences, and the same letter indicates no significant difference.

Relative electric conductivity is an important indicator of cell membrane permeability. As shown in Figure 3A, the relative electric conductivity was significantly lower by 11.30% and 21.45%, respectively, for loquat fruit treated with 0.05% L-Cys and 0.05% GABA compared to the control (*p* < 0.05). These results indicated that L-Cys and GABA treatments inhibited the increase in relative electric conductivity for better maintenance of the integrity of the cell membrane.

Malondialdehyde (MDA) measurement indicates the level of lipid peroxidation in the cell membrane. As shown in Figure 3B, treatments of 0.05% L-Cys and 0.05% GABA resulted in MDA values 28.99% and 40.95% lower than the control at 40 days of storage, respectively (*p* < 0.05). This showed that L-Cys and GABA treatments effectively inhibited the increase in MDA content at 40 days of storage and reduced the accumulation of harmful substances that damage cells, resulting in better quality of stored loquat fruit.

### 2.5. Effects of L-Cys and GABA Treatments on Total Phenols, Total Flavonoids, Flavanols, and Carotenoids in Loquat Fruit after Harvest

**Figure 4:**
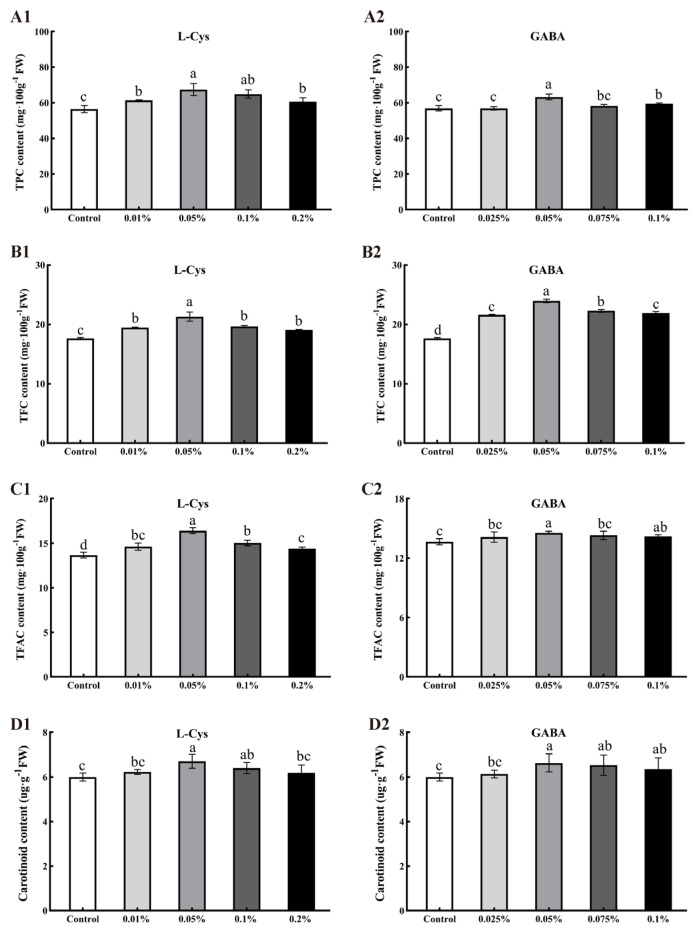
Effects of different concentrations of L-Cys and GABA treatments on TPC (**A**), TFC (**B**), TFAC (**C**), and carotenoid content (**D**) of loquat fruit after 40 days of storage. A1, B1, C1, D1 and A2, B2, C2, D2 indicate the effect of L-Cys and GABA, respectively. Each column represents mean ± standard deviation (n = 3), and a *t* test was performed at the 0.05 significance level (*p* < 0.05). Different letters (a, b, c, d) indicate significant differences, and the same letter indicates no significant difference.

As shown in Figure 4A, the total phenolic content measurements for loquat fruit treated with 0.05% L-Cys, 0.1% L-Cys, 0.05% GABA, and 0.025% GABA were 19.46%, 15.05%, 12.11%, and 5.41%, respectively, higher compared to the control. Thus, L-Cys and GABA treatments maintained the total phenolic content of loquat fruit and significantly inhibited its decrease (*p* < 0.05). Among treatments, 0.05% L-Cys and 0.05% GABA treatment had the best effect.

As seen in Figure 4B, the TFC content was higher in loquat fruits treated with different concentrations of L-Cys and GABA compared to the control. The total flavonoid content of loquat fruits treated with 0.05% L-Cys and 0.05% GABA were highest at 40 days of storage, 16.67% and 33.33% higher than that in the control. L-Cys and GABA treatments significantly inhibited the decrease of total flavonoid content (*p* < 0.05).

As shown in Figure 4C, the total flavanol content measurements of loquat fruit treated with 0.05% L-Cys, 0.1% L-Cys, and 0.05% GABA were 19.71%, 9.49%, and 6.57% higher than that in the control. Of the different treatments, 0.05% L-Cys and 0.05% GABA had the best effect (*p* < 0.05).

At 40 days of storage, carotenoid content was significantly higher in loquat fruit treated with 0.05% L-Cys and 0.05% GABA than in the control (*p* < 0.05) by 11.67% and 10.5%, respectively (Figure 4D). The application of L-Cys and GABA significantly slowed the decrease of carotenoids in loquat during storage.

### 2.6. Effects of L-Cys and GABA on DPPH and FRAP of Loquat Fruit after Harvest

**Figure 5:**
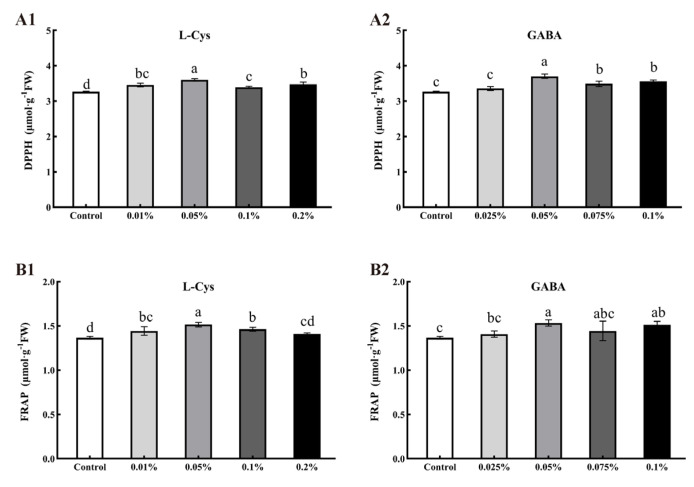
Effects of different concentrations of L-Cys and GABA treatments on DPPH (**A**) and FRAP (**B**) of loquat fruit after 40 days of storage. A1, B1 and A2, B2 indicate the effect of L-Cys and GABA, respectively. Each column represents mean ± standard deviation (n = 3), and a *t* test was performed at the 0.05 significance level (*p* < 0.05) Different letters (a, b, c, d) indicate significant differences, and the same letter indicates no significant difference.

L-Cys and GABA treatments significantly maintained the scavenging ability of DPPH free radicals in loquat fruits after 40 days of storage (Figure 5A). The DPPH scavenging capacity in loquat fruits treated with 0.05% L-Cys and 0.05% GABA were 3.598 μmol·g^−1^FW and 3.704 μmol·g^−1^FW, i.e., 10.0% and 13.15% higher than the capacity of the control (*p* < 0.05).

As shown in Figure 5B, L-Cys and GABA treatments promoted the FRAP in loquat fruit after 40 days of storage. Treatments of 0.05% L-Cys and 0.05% GABA significantly promoted the FRAP of loquat fruit, 10.65% and 11.68% higher than that of the control (*p* < 0.05), for improved antioxidant capacity.

### 2.7. Effects of L-Cys and GABA on SOD, CAT, POD, and PPO Activity of Loquat Fruit after Harvest

**Figure 6:**
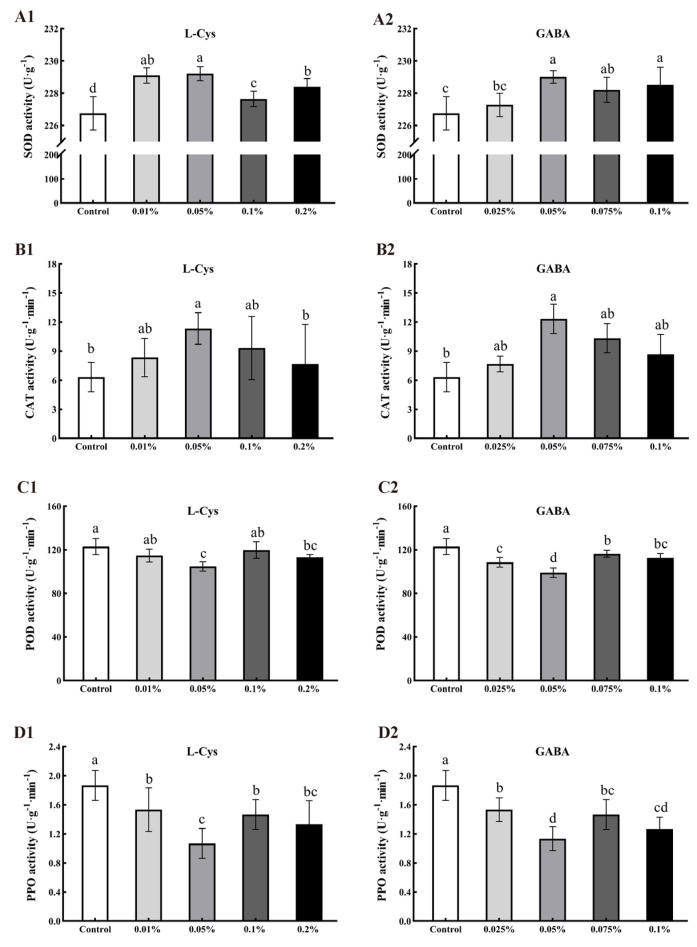
Effects of different concentrations of L-Cys and GABA treatments on SOD (**A**), CAT (**B**), POD (**C**) and PPO activity (**D**) of loquat fruit after 40 days of storage. A1, B1, C1, D1 and A2, B2, C2, D2 indicate the effect of L-Cys and GABA, respectively. Each column represents mean ± standard deviation (n = 3), and a *t* test was performed at the 0.05 significance level (*p* < 0.05). Different letters (a, b, c, d) indicate significant differences, and the same letter indicates no significant difference.

SOD and CAT are antioxidant enzymes that scavenge active oxygen free radicals in fruit. As shown in Figure 6A, L-Cys and GABA treatments allowed maintenance of high SOD activity after 40 days of loquat fruit storage, with 0.05% L-Cys, 0.05% GABA, and 0.1% GABA treatments significantly higher than the control (*p* < 0.05). After 40 days of loquat fruit storage, the CAT content of the control group (Figure 6B) was significantly lower than that of other treatment groups. Treatments with 0.05% L-Cys and 0.05% GABA were 78.95% and 94.74% higher than the control group, respectively, indicating that these treatments allowed maintenance of higher CAT activity in loquat fruit.

POD can catalyze the oxidative polymerization of phenols in fruits, resulting in browning and lignin synthesis. Treatments with 0.05% L-Cys and 0.05% GABA resulted in 17.39% and 24.21% decreases in POD activity, respectively (*p* < 0.05). The effect of L-Cys and GABA treatments to decrease the POD activity of loquat fruit reduced the incidence of fruit decay and browning.

PPO is a multifunctional enzyme widely found in fruits and vegetables, acting to catalyze the oxidation of polyphenols into quinones and cause browning of fruits and vegetables. As shown in Figure 6D, the PPO activity of treated loquat fruit was lower than that of the control group after 40 days of storage, with 0.05% L-Cys and 0.05% GABA treatment reducing the PPO activity of loquat fruit by 74.98% and 64.71%, respectively (*p* < 0.05). Thus, L-Cys and GABA treatments delayed the increase of PPO activity during storage of loquat fruit to inhibit fruit browning.

### 2.8. PCA 

**Figure 7:**
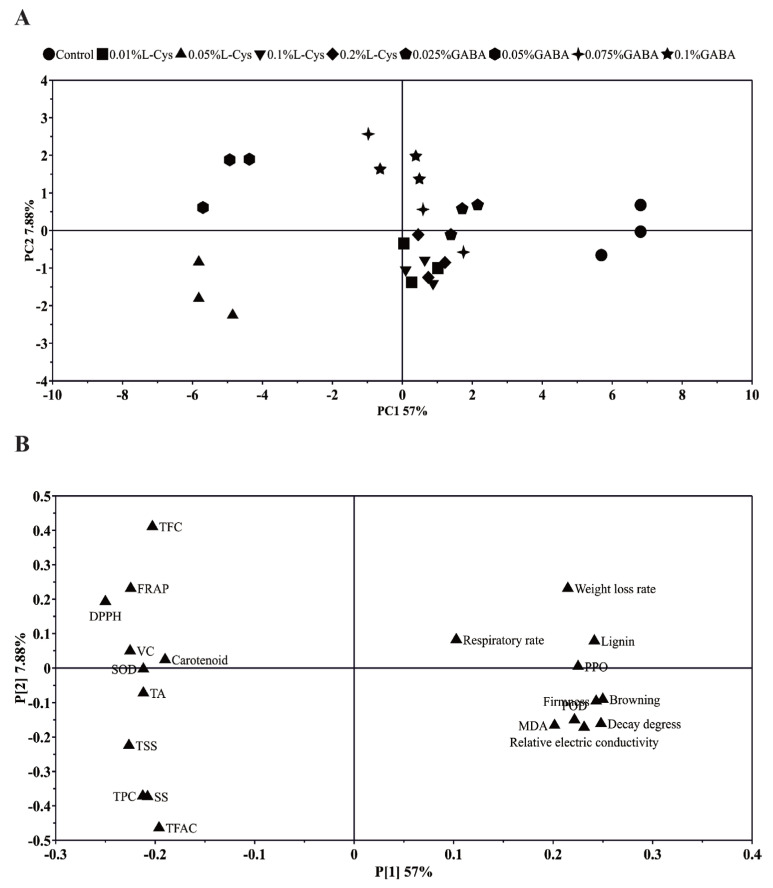
Principal component analysis of postharvest storage quality of loquat: (**A**) principal component score map; (**B**) PCA loading diagram.

Principal component analysis (PCA) was used to explore the effects of two postharvest treatments on loquat quality. Two principal components (PCs) explained 64.88% of the total variance. PC1 and PC2 accounted for 57% and 7.88% of the total variance, respectively. Treatments of 0.05% L-Cys and 0.05% GABA were significantly different from other groups along the PC1 axis and loaded along the negative direction of PC1. The control group was significantly separated from L-Cys and GABA-treated samples, with no significant separation between the other treated samples (Figure 7A). Loquat fruits stored for 40 days under L-Cys and GABA treatments could be distinguished by PC1. PC1 was positively correlated with some quality parameters such as weight loss rate, lignin content, respiratory rate, and PPO, and negatively correlated with fruit Vc, carotenoids, DPPH, FRAP, TFC, and CAT (Figure 7B). Overall, the results clearly indicate that 0.05% L-Cys and 0.05% GABA treatments can maintain the appearance and internal quality of loquat fruit.

### 2.9. Analysis of Relationships

**Figure 8:**
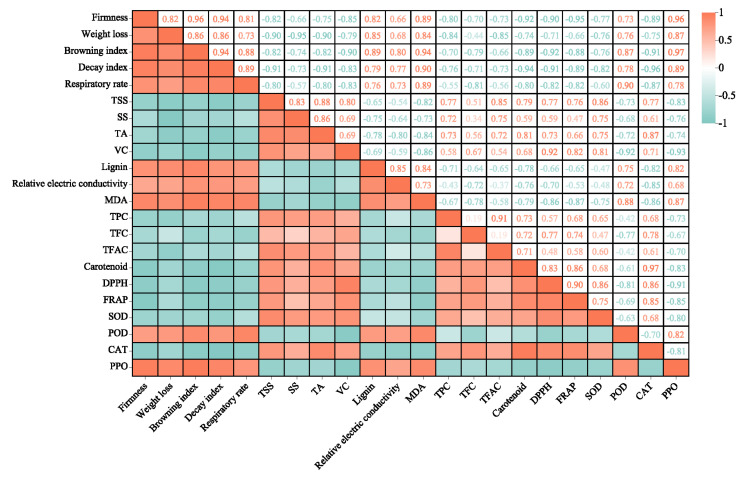
Correlation of fruit parameters during cold storage of loquat treated with L-Cys and GABA. The numerical value and color intensity are proportional to the correlation coefficient. Orange indicates a positive correlation, and green indicates a negative correlation (*p* < 0.05).

Pearson correlation coefficient analysis was used to analyze the correlation between appearance quality, internal quality, phenolic substances, and antioxidant enzyme activity during loquat cold storage. Significant positive (orange) and negative (green) correlations are shown in Figure 8. The firmness was positively correlated with weight loss rate, browning index, decay index, respiration rate, relative electric conductivity, lignin content, MDA, POD, and PPO, while TPC, TFC, TFAC, DPPH, and FRAP were negatively correlated with MDA, POD, PPO, and lignin.

## 3. Discussion

**Figure 9:**
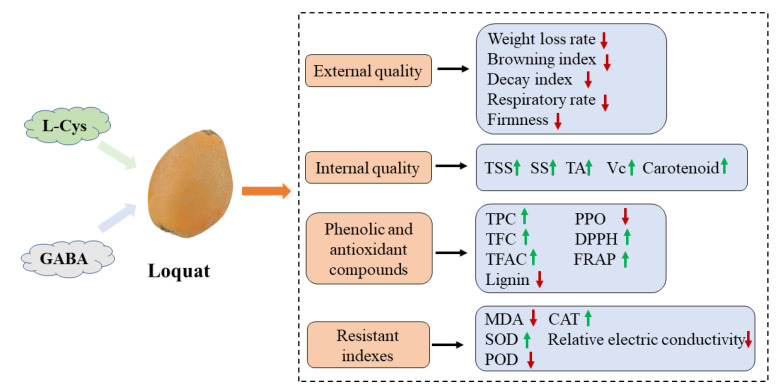
Changes in postharvest quality and antioxidant system of loquat fruit treated with L-Cys and GABA under cold storage conditions. The green arrow indicates an increase, and the red arrow indicates a decrease.

Loquat is popular for its delicious taste and high levels of dietary antioxidants (such as carotenoids and flavonoids) and other bioactive compounds. However, due to the thin and juicy peel of loquat fruit, its post-harvest quality deteriorates rapidly. This is mainly manifested as dehydration shrinkage, decay, browning, and lignification, seriously reducing the commercial value of loquat fruit. L-Cys and GABA are important exogenous amino acids that play key roles in plant growth and stress response. This study examined the effects of L-Cys and GABA treatments on fruit quality and the antioxidant system of loquat during cold storage (Figure 9).

Weight loss rate, browning index, and decay index are important indicators reflecting the effect of fruit storage. The results showed that 0.05% L-Cys and 0.05% GABA treatments effectively reduced the weight loss rate, browning index, and decay index of loquat fruit compared with other treatments. Ali et al. [21] similarly found that 0.25% L-Cys can effectively reduce the weight loss rate and delay the browning index of litchi. Fan et al. [22] found that 1.0 mM GABA could reduce the weight loss rate of olive fruit during cold storage. Firmness is an important quality characteristic, and can defend against mechanical damage to improve the shelf life of fruits. However, during the postharvest storage of loquat fruit, the increase in firmness accompanied by the accumulation of lignin content reduces the flavor of the fruit [23]. Loquat treated with 0.05% L-Cys and 0.05% GABA exhibited a significantly delayed increase in firmness. This was consistent with previous findings [24] that the combined application of oxalic acid (OA) and modified vapor phase packaging (MAP) delayed the firmness and shelf life of loquat. Respiration rate is a good physiological index to evaluate the energy metabolism of postharvest fruit. This study found that 0.05% L-Cys and 0.05% GABA treatments significantly inhibited the respiration rate of loquat fruit after harvest, but the detailed mechanisms explaining the effects of L-Cys and GABA treatments on postharvest fruit respiration are still unclear. GABA can accumulate in *Arabidopsis* mutants lacking respiratory enzymes, indicating a key role of GABA in plant respiration [25]. Ngaffo et al. [26] found that the interaction between GABA and postharvest fruit CO_2_ concentration indicated that GABA helped to reduce the respiratory activity of carambola fruit during cold storage.

Intrinsic quality is an important index that affects the taste, flavor, and nutrition of fruits, and the content of intrinsic substances in fruits gradually decreases with fruit aging [27,28]. L-Cys and GABA treatments can improve the intrinsic quality of fruits and vegetables, and in this work, loquat fruits treated with 0.05% L-Cys and 0.05% GABA maintained fruit quality better than the control treatment. Studies have found that L-Cys treatment can significantly delay the decline of soluble sugar, titratable acid, and carotenoids in litchi fruits. Other work also showed that L-Cys is a sulfur-containing amino acid that can delay fruit senescence [21,29]. Another study on olive found that 1.0 mM GABA significantly increased the soluble solids, soluble sugar, titratable acid and chlorophyll content of olive fruit [22]. Vc is related to anti-oxidation and anti-free radical [30]. This study showed that 0.05% L-Cys and 0.05% GABA treatments significantly delayed the decrease in Vc, which was consistent with the results of Sohail et al. [31].

Phenols, flavonoids, flavanols, carotenoids, and Vc in Loquat are free radical scavengers that protect important molecules in cells from oxidation. The indexes related to tissue antioxidant capacity include DPPH free radical scavenging activity and FRAP [32]. Phenolic compounds are important secondary metabolites of plants, which are essential to defend against postharvest pathogens in fruits, and have strong antioxidant activities, which can improve the fruit nutritional value [33,34]. Carotenoid is the main determinant of loquat fruit color, and its content is highest in fruit. This study found that compared with the control, 0.05% L-Cys and 0.05% GABA treatments could significantly increase the phenols and antioxidants of loquat fruit. This was similar to the conclusion of Ebrahimzadeh et al. [35] that 1% gum arabic (rich in 5.0 mM GABA) significantly increased the total phenolic content of walnut kernels and delayed the decrease in antioxidant capacity. Postharvest studies of bananas also found that GABA treatment promoted the increase in total phenol content in banana fruits during storage [19]. The DPPH free radical scavenging activity and FRAP results on the antioxidant capacity of loquat fruit were consistent with the total phenol content. Treatments of 0.05% L-Cys and 0.05% GABA treatment significantly delayed the decrease in DPPH and FRAP in loquat fruit at 40 d. Preczenhak et al. [36] showed that Cys treatment could improve the antioxidant capacity of fresh-cut red beet and lettuce.

In recent years, studies have shown that the lignin biosynthesis pathway is made up of the phenylpropanoid metabolic and lignin synthesis specific pathways. PPO and POD are key enzymes in lignin synthesis, providing structural support for plant growth, development, and defense [37,38]. PPO and POD can oxidize phenols, which are mainly involved in the browning of fruits and vegetables during cold storage. PPO is an important factor that causes enzymatic browning of fruits and vegetables, leading to spoilage [39]. POD is an antioxidant enzyme and can eliminate peroxide free radicals, ensure the integrity of the membrane system, and maintain normal metabolism [40]. Treatments of 0.05% L-Cys and 0.05% GABA significantly inhibited the activity of lignin, PPO, and POD in loquat fruit, effects that are closely related to the reduction in fruit browning rate. Sun et al. [41] showed that NO delayed the formation and inhibited the accumulation of okra lignin. Cai et al. [42] reported that low temperature storage could inhibit the activities of PPO and POD in loquat fruit, thereby reducing flesh browning. Studies have also shown that light (2000 lux) treatment significantly inhibited the PPO and POD activities of fresh-cut celery, thereby reducing the change in the browning index during storage [43].

The relative conductivity and MDA are important indicators of cell membrane permeability; the greater the value, the greater is the damage to the tissue cell membrane. In this study, L-Cys and GABA treatments reduced the relative conductivity of loquat fruit and inhibited the accumulation of MDA, which indicated that L-Cys and GABA had a positive effect on maintaining the integrity and stability of the cell membrane of postharvest loquat fruit. L-Cys and GABA reduced the chilling injury of postharvest loquat fruit by protecting the cell membrane structure. This is similar to the conclusion of Shekari et al. [44] that exogenous GABA inhibits MDA accumulation during low temperature storage of mushrooms. There are antioxidant enzyme systems in fruit and vegetable tissues. SOD and CAT are the main enzymes that scavenge reactive oxygen species in plant tissues. The coordination of these two enzymes can maintain reactive oxygen species at a low level, thereby preventing their damage to cells [45]. Loquat is a cold-sensitive fruit. When cold-sensitive fruit is stored at an unsuitable low temperature, the cell membrane first responds to low-temperature stress, reducing the activity of the oxygen-scavenging enzyme system, resulting in the disorder of active oxygen metabolism in the fruit. Excessive accumulation of reactive oxygen species in fruit tissue will accelerate membrane lipid peroxidation and eventually leads to fruit damage [46]. This study found that 0.05% L-Cys and 0.05% GABA treatments could significantly increase the activities of SOD and CAT in loquat fruit. Studies on cucumber have found that fucoidan treatment can increase SOD and CAT activity [47]. Treatment with 10 μmol·L^−1^ MeJA could increase the activities of SOD and CAT, reduce the accumulation of reactive oxygen species, and alleviate the symptoms of lignification and chilling injury caused by low temperature [48]. Overall, the results indicate that L-Cys and GABA post-harvest treatments delayed the browning and senescence of loquat fruit mainly by improving the integrity of the cell membrane and antioxidant enzyme activity during cold storage.

In summary, treatments with 0.05% L-Cys and 0.05% GABA during loquat storage resulted in an increase in the content of bioactive compounds in the fruits. The treatments with L-Cys and GABA also significantly reduced the weight loss rate, browning index, decay index, and respiratory intensity of the loquat. In addition, the treatments helped to maintain higher levels of TSS, SS, TA, Vc, and carotenoid contents; improved phenolic substances and antioxidant capacity; and delayed the increase in fruit firmness, relative electric conductivity, MDA, PPO, and POD. In general, our results show that 0.05% L-Cys and 0.05% GABA treatment are effective methods to alleviate the decay and browning of loquat fruit and maintain fruit quality during cold storage.

## 4. Materials and Methods

### 4.1. Plant Materials and Treatments

‘Zaozhong No.6’ loquat fruit were harvested in Sichuan Province, China (26°42′ N, 101°44′ E), at an altitude of 2080 m on 17 March 2022. Loquat fruits without disease and damage were selected and packed in a protective bag and immediately transported to the laboratory of Sichuan Agricultural University. Loquat fruits (n = 270) were randomly divided into nine groups with three replicates in each group (each replicates includes ten fruits). The nine groups of fruit were dipped in water (control), L-Cys (0.01%, 0.05%, 0.1%, 0.2%), or GABA (0.025%, 0.05%, 0.075%, 0.1%) solutions for 10 min (L-Cysteine and γ-aminobutyric acid were dissolved in distilled water). After the fruit were air-dried, they were put into PE film bags and stored at 4 ± 0.5 °C and 90% relative humidity for 35 days. The weight loss, browning index, decay index, and respiration rate were measured every 7 days. After cold storage, the fruit were stored under a simulated shelf-life environment (22 °C ± 0.5 °C, 85% relative humidity) for 5 days and then the quality was evaluated. At the same time, samples were frozen, chilled in liquid nitrogen, and stored at −80 °C.

### 4.2. Determination of Weight Loss Rate, Browning Index, Decay Index, and Respiratory Rate

Weight loss rate, browning index, and decay index were estimated according to the method of Cao et al. [49]. To determine the weight loss of individual loquat fruit, the fruit was weighed before storage (initial weight) and after each sampling date (final weight). The weight loss was then calculated and expressed as a percentage loss, using the equation: weight loss rate (%) = (quality before storage – quality after storage) 100/quality before storage. The browning index and decay index were calculated by grading method. The browning index is assessed by measuring the extent of the total area of brown tissue flesh of each fruit on the following scale: 0 = No browning; 1 = Slight browning (<5%); 2 = Moderate browning (5–25%); 3 = Moderate severe browning (25–50%); 4 = Severe browning (>50%). The browning index (%) was calculated as ∑ (browning grade × number of browning fruits at all levels) / (total number of fruits × highest grade) × 100. Decay index is assessed by measuring the extent of the total area of decayed tissue of each fruit on the following scale: 0 = No decay; 1 = Slight decay (<10%); 2 = Moderate decay (10–30%); 3 = Moderate severe decay (30–50%); 4 = Severe decay (≥50%). The decay index (%) was calculated as ∑ (decay grade × number of decay fruits at all levels)/(total number of fruits × highest grade) × 100. The respiration rate of loquat was measured by the method of Chen et al. [50] with slight modifications. The fruits were placed in a sealed tank for 1 h, and then the CO_2_ content in the tank was measured. The results were expressed as mg·kg^−1^·h.

### 4.3. Determination of Total Soluble Solids (TSS), Soluble Sugar (SS), Titratable Acid (TA) and Vitamin C (Vc) Content

The amount of total soluble solids was determined by digital refractometer, and the values were averaged from three points on the equatorial line of the fruit. The content of soluble sugar in pulp was determined by anthrone colorimetry [51]. Titratable acid in pulp was determined by sodium hydroxide titration [52]. The vitamin C content in pulp was determined by the Fe^3+^ reduction method [53].

### 4.4. Determination of Fruit Firmness and Lignin

The firmness was measured with a WDGY-4 fruit firmness tester. The extraction and content determination of lignin were performed according to the method of Femenia [54] The absorbance value was measured at 280 nm, and lignin content was expressed as %.

### 4.5. Determination of Relative Electric Conductivity and MDA

The relative electric conductivity (EC) was measured with a conductivity meter [55]. To do this, 1 g of flesh was weighed and added to a 50 mL centrifuge tube containing 20 mL of sterile water. The mixture was oscillated for 30 min, and the first conductivity was measured. The sample was then boiled for 10 min, allowed to cool to room temperature, and the final conductivity was measured.

The content of malondialdehyde was determined by thiobarbituric acid reaction [56]. First, 0.5 g tissue was mixed in 5 mL phosphate buffer (pH = 7.8) and centrifuged at 4500× *g* for 10 min. Then, 3 mL of 0.5% thiobarbituric acid (containing 20% TCA) was added to 2 mL of the supernatant. The mixture was incubated at 100 °C for 10 min, and immediately cooled before reading the absorbance at 532 and 600 nm, and the results were expressed as nmol·g^−1^.

### 4.6. Determination of Total Phenolic Content

Total phenolic content (TPC) was determined by the method of Zhang et al. [57]. The total phenols were extracted with 70% methanol (containing 2% formic acid), and the supernatant was mixed with Folin–Ciocalteu reagent. After 1 min, 20% saturated sodium carbonate was added and mixed well. The absorbance was measured at 760 nm with a spectrophotometer, and gallic acid was used as the standard (50–1000 mg·L^−1^).

### 4.7. Determination of Flavonoid Content

The content of total flavonoids (TFC) was determined according to the method of Zhang et al. [57]. The total flavonoids were extracted with 70% methanol (containing 2% formic acid), and the supernatant was mixed with methanol, sodium nitrite, and aluminum chloride reagent, and then mixed with sodium hydroxide after 5 min. The absorbance was measured at 510 nm by spectrophotometer. Rutin was used as the standard (20–100 mg·L^−1^).

### 4.8. Determination of Total Flavanol Content

The content of flavanols (TFAC) was determined according to the method of Zhang et al. [57]. The total flavonoids were extracted with 70% methanol (containing 2% formic acid), and the supernatant was mixed with distilled water and 1 mL 1% p-DMACA solution. After 10 min, the absorbance was measured by spectrophotometer at 640 nm, and catechin was used as the standard (6.25–200 mg·L^−1^).

### 4.9. Determination of Carotenoids

The carotenoid content of loquat fruit was measured by the 95% ethanol extraction method [58]. The loquat sample was soaked in ethanol (95%) until colorless. The result was indicated as µg·g^−1^ FW.

### 4.10. Determination of DPPH and FRAP

The DPPH scavenging ability was determined by the method of Brand-Williams et al. [59]. The extracted sample was added to distilled water and DPPH methanol solution, and the absorbance was measured at 517 nm by spectrophotometer after 20 min reaction in the dark.

FRAP was determined using the method of Benzie [60] with minor modifications. The extracted sample was added to distilled water and iron reduction antioxidant power (TPTZ) working solution, and reacted at 37 ° C for 10 min before quickly cooling to room temperature. The absorbance was recorded at 593 nm by spectrophotometer.

### 4.11. Determination of Superoxide Dismutase (SOD), Catalase (CAT), Peroxidase (POD), and Polyphenol Oxidase (PPO) Activity

Frozen samples of approximately 0.25 g were quickly weighed and added to 5 mL ice-cold phosphate buffer (containing 1 g PVP, 0.003085 g DTT, and 0.0037224 g EDTA), centrifuged at 8000 rpm for 15 min at 4 °C, and the supernatant was used as the crude enzyme extract to measure four enzyme activity indicators.

The method of Rao et al. [61] was used to measure SOD, with slight changes. The reaction mixture (3 mL) contained 50 mmol·L^−1^ sodium phosphate buffer (pH = 7.8), 130 mmol·L^−1^ methionine, 750 mmol·L^−1^ nitroblue tetrazolium (NBT), 100 μmol·L^−1^ EDTA, 20 μmol·L^−1^ riboflavin, and 0.1 mL enzyme extract. By recording the absorbance at 560 nm, the inhibition of NBT photoreduction by 50% at 560 nm wavelength per minute per gram of flesh was determined as an enzyme activity in units of U·g^−1^.

CAT activity was measured according to Aghdam et al. [62] with minor modifications. The reaction mixture contained 0.15 M (pH = 7.0) phosphate buffer and 30% H_2_O_2_, and was quickly added to 0.1 mL of extracted sample and shaken. The absorbance change at 240 nm wavelength was measured, and the absorbance change at 240 nm per minute was defined as 0.01 CAT activity unit. The results were expressed as U·g^−1^·min^−1^.

POD activity was determined using the guaiacol method [63] with slight modifications. The reaction mixture contained 0.2 M (pH = 6.0) phosphate buffer, 2% H_2_O_2_, 0.05 mol·L^−1^ guaiacol, and 0.5 mL enzyme solution mixed well. The change in absorbance value at 470 nm was measured, and a 0.01 change in absorbance value per minute at 470 nm was defined as one POD activity unit. The results were expressed as U·g^−1^·min^−1^ of enzyme activity.

PPO was determined by the catechol method [64] with slight modifications. The reaction mixture contained 0.2 M (pH = 7.8) phosphate buffer and 0.05 mol·L^−1^ catechol, which was quickly added to 0.5 mL of crude enzyme extract and shaken. The absorbance change was immediately measured at 410 nm wavelength. A 0.01 increase per minute in absorbance value was defined as a POD activity unit, and the results were expressed as U·g^−1^·min^−1^.

### 4.12. Statistical Analysis

All data are expressed as mean ± standard error (SE) (n = 3) in this paper. The data analysis was performed with IBM SPSS 26.0 statistical software for analysis of variance (ANOVA). Duncan’s multiple range test was used to determine the differences between treatments at a significance level of *p* < 0.05. Principal component analysis (PCA) was performed using SIMCA 14.0 software.

## Figures and Tables

**Table 1:** Effects of different concentrations of L-Cys and GABA treatments on weight loss rate, browning index, decay index, and respiratory rate of loquat during storage. Values of three replicates are expressed as the means ± SD. Different lowercase letters (a–c) in the table denote significant differences between sampling dates for each treatment by Duncan’s multiple range test (*p* < 0.05).

Storage Time									
	Treatments	Concentration	0 Day	7 Days	14 Days	21 Days	28 Days	35 Days	40 Days
Weight loss rate (%)	Control	Water	0	0.77 ± 0.23a	1.21 ± 0.18a	1.62 ± 0.10a	2.54 ± 0.12a	3.51 ± 0.05a	4.74 ± 0.12a
	L-Cys	0.01%	0	0.58 ± 0.10a	0.94 ± 0.04a	1.40 ± 0.08ab	1.75 ± 0.08cd	3.15 ± 0.11abc	4.29 ± 0.20bc
		0.05%	0	0.45 ± 0.09a	0.87 ± 0.09a	1.10 ± 0.02c	1.56 ± 0.10d	2.94 ± 0.06bc	3.91 ± 0.05c
		0.1%	0	0.54 ± 0.05a	1.11 ± 0.06a	1.37 ± 0.04b	2.15 ± 0.13b	3.10 ± 0.14abc	4.32 ± 0.10abc
		0.2%	0	0.65 ± 0.09a	1.18 ± 0.09a	1.43 ± 0.05ab	2.22 ± 0.08b	3.19 ± 0.15abc	4.27 ± 0.12bc
	GABA	0.025%	0	0.62 ± 0.04a	1.03 ± 0.09a	1.21 ± 0.11bc	2.19 ± 0.13b	3.28 ± 0.11ab	4.39 ± 0.12ab
		0.05%	0	0.41 ± 0.06a	0.89 ± 0.09a	1.19 ± 0.03bc	1.82 ± 0.01cd	2.86 ± 0.11c	4.10 ± 0.18bc
		0.075%	0	0.66 ± 0.14a	1.00 ± 0.14a	1.38 ± 0.04b	2.18 ± 0.06b	3.05 ± 0.19bc	4.44 ± 0.09ab
		0.1%	0	0.64 ± 0.02a	1.10 ± 0.11a	1.36 ± 0.10b	1.20 ± 0.03bc	3.13 ± 0.13abc	4.51 ± 0.09ab
Browning index (%)	Control	Water	0	7.61 ± 1.18a	11.11 ± 1.39a	27.08 ± 2.08a	44.17 ± 0.17a	60.42 ± 5.51a	71.00 ± 2.02a
	L-Cys	0.01%	0	5.55 ± 2.78ab	4.83 ± 0.35c	18.89 ± 1.94bcd	38.33 ± 0.83ab	55.83 ± 1.27ab	61.83 ± 2.04b
		0.05%	0	2.78 ± 1.47ab	4.10 ± 0.54c	15.56 ± 0.56d	28.33 ± 1.67c	38.89 ± 2.00c	49.83 ± 0.63c
		0.1%	0	6.93 ± 1.40ab	7.50 ± 1.73abc	19.16 ± 1.27bcd	35.28 ± 1.21b	53.06 ± 1.55ab	59.28 ± 1.62b
		0.2%	0	3.15 ± 1.61ab	8.89 ± 0.56ab	21.94 ± 1.55bc	42.22 ± 1.47a	52.78 ± 2.78ab	59.17 ± 2.20b
	GABA	0.025%	0	5.26 ± 1.57ab	7.07 ± 1.26bc	17.78 ± 1.11cd	39.72 ± 1.21ab	54.72 ± 2.90ab	60.53 ± 2.22b
		0.05%	0	1.67 ± 1.67b	5.56 ± 1.39bc	15.56 ± 0.56d	28.06 ± 1.55c	38.33 ± 0.83c	48.17 ± 1.41c
		0.075%	0	3.04 ± 1.54ab	6.67 ± 1.67bc	20.00 ± 0.00bcd	35.00 ± 2.89b	46.67 ± 1.67bc	58.33 ± 1.67b
		0.1%	0	3.04 ± 1.54ab	6.06 ± 0.97bc	23.33 ± 1.67ab	41.67 ± 4.41ab	50.56 ± 4.44b	57.72 ± 0.96b
Decay index (%)	Control	Water	0	5.14 ± 0.60a	12.44 ± 1.44a	20.00 ± 2.89a	30.28 ± 4.72a	35.33 ± 2.17a	49.36 ± 1.78a
	L-Cys	0.01%	0	2.10 ± 1.20ab	9.72 ± 1.39abc	13.33 ± 0.83bc	23.33 ± 2.93abc	28.05 ± 3.74bc	42.00 ± 1.75b
		0.05%	0	0.72 ± 0.72b	6.93 ± 1.40c	11.11 ± 2.00c	20.56 ± 2.42 bc	26.67 ± 1.67bc	34.17 ± 2.20c
		0.1%	0	0.72 ± 0.72b	11.06 ± 0.82 ab	14.72 ± 1.21abc	23.33 ± 1.67abc	30.83 ± 1.27abc	42.67 ± 0.51ab
		0.2%	0	2.39 ± 1.45ab	9.61 ± 0.66abc	14.17 ± 0.83abc	23.33 ± 1.67abc	32.78 ± 1.47ab	44.50 ± 2.30ab
	GABA	0.025%	0	4.45 ± 0.28a	10.28 ± 1.21abc	11.67 ± 0.83c	25.28 ± 2.65abc	31.11 ± 1.94abc	45.61 ± 1.22ab
		0.05%	0	0.72 ± 0.72b	7.55 ± 1.68bc	10.83 ± 0.83c	18.89 ± 1.11c	25.00 ± 0.00c	34.44 ± 0.56c
		0.075%	0	3.33 ± 1.67ab	10.00 ± 0.00abc	16.67 ± 1.67abc	28.44 ± 2.51ab	30.00 ± 2.89abc	40.83 ± 1.59bc
		0.1%	0	3.05 ± 1.55ab	11.58 ± 0.80a	18.72 ± 3.20ab	23.61 ± 1.39abc	30.83 ± 1.27abc	42.22 ± 1.47b
Respiratory rate (mg·kg^−1^·h)	Control	Water	83.24 ± 7.96	81.57 ± 1.58a	79.81 ± 0.68a	76.78 ± 1.07a	73.45 ± 1.11a	70.54 ± 0.72a	67.05 ± 0.85a
	L-Cys	0.01%	83.24 ± 7.96	79.00 ± 3.19abc	78.04 ± 3.78ab	75.54 ± 1.18a	71.29 ± 1.22ab	68.94 ± 0.60a	66.03 ± 0.78ab
		0.05%	83.24 ± 7.96	73.83 ± 1.36bc	72.67 ± 0.52ab	68.26 ± 1.40b	67.54 ± 0.69bc	64.84 ± 0.34b	63.04 ± 0.25c
		0.1%	83.24 ± 7.96	78.36 ± 2.02abc	72.64 ± 4.04ab	76.16 ± 0.70a	72.23 ± 1.24a	69.50 ± 0.62a	66.19 ± 1.62ab
		0.2%	83.24 ± 7.96	79.30 ± 0.61abc	79.62 ± 3.69aab	74.60 ± 1.45a	72.98 ± 1.20a	70.31 ± 1.50a	66.90 ± 0.85a
	GABA	0.025%	83.24 ± 7.96	80.32 ± 2.86ab	78.45 ± 1.19a	74.28 ± 2.28a	72.00 ± 1.20a	70.55 ± 1.45a	65.20 ± 0.49abc
		0.05%	83.24 ± 7.96	72.20 ± 2.01c	70.44 ± 1.53b	67.81 ± 1.47b	66.10 ± 1.43b	63.70 ± 1.10b	62.33 ± 0.37bc
		0.075%	83.24 ± 7.96	77.75 ± 1.94abc	75.82 ± 0.62ab	78.47 ± 2.46a	72.60 ± 1.65a	71.85 ± 0.65a	65.62 ± 1.35ab
		0.1%	83.24 ± 7.96	78.59 ± 2.67abc	78.42 ± 0.44a	75.68 ± 0.50a	72.14 ± 1.71a	70.70 ± 0.90a	65.35 ± 1.19abc

## Data Availability

The data and materials supporting the conclusions of this study are included within the article.
